# Optimal Parameters of Laser Therapy to Improve Critical Calvarial Defects

**DOI:** 10.3389/fphys.2022.841146

**Published:** 2022-02-25

**Authors:** Matheus AFM Santos, Daniela N. Silva, Karla Rovaris, Frederico B. Sousa, Eugenia LA Dantas, Lucas A. Loureiro, Thiago M. C. Pereira, Silvana S. Meyrelles, Rossiene M. Bertollo, Elisardo C. Vasquez

**Affiliations:** ^1^Dentistry Graduate Program, Federal University of Espirito Santo, UFES, Vitoria, Brazil; ^2^Department of Pathology & Clinical Dentistry, Federal University of Piaui, Teresina, Brazil; ^3^Department of Morphology, Federal University of Paraiba, UFPB, Joao Pessoa, Brazil; ^4^Dentistry Graduate Program, UFPB, Joao Pessoa, Brazil; ^5^Pharmaceutical Sciences Graduate Program, Vila Velha University, Vila Velha, Brazil

**Keywords:** calvarial defect, IBB, blood clot, laser therapy, brain function

## Abstract

Body bones play diverse pivotal roles, including the protection of vital organs. For instance, the integrative functions of the brain controlling diverse peripheral actions can be affected by a traumatic injury on the calvaria and the reparative process of a large defect is a challenge in the integrative physiology. Therefore, the development of biomaterials and approaches to improve such defects still requires substantial advances. In this regard, the most attractive approaches have been covering the cavity with inorganic bovine bone (IBB) and, more recently, also using low-level laser therapy (LT), but this issue has opened many questions. Here, it was determined the number of LT sessions required to speed up and to intensify the recovery process of two 5-mm-diameter defects promoted in the calvaria of each subgroup of six adult Wistar rats. The quantitative data showed that 30 days post-surgery, the recovery process by using blood clot-filling was not significantly influenced by the number of LT sessions. However, in the IBB-filled defects, the number of LT sessions markedly contributed to the improvement of the reparative process. Compared to the Control group (non-irradiated), the percentage of mineralization (formation of new bone into the cavities) gradually increased 25, 49, and 52% with, respectively, 4, 7, and 11 sessions of LT. In summary, combining the use of IBB with seven sessions of LT seems to be an optimal approach to greatly improve the recovery of calvarial defects. This translational research opens new avenues targeting better conditions of life for those suffering from large bone traumas and in the present field could contribute to preserve the integrative functions of the brain.

## Introduction

Body bones play a pivotal role, protecting the vital organs, articulating body movements, producing blood cells, and contributing to calcium homeostasis. A characteristic of this tissue is that when injured it heals without scarring and, despite exhibiting cellular machinery and all components to initiate a necessary regenerative process, there are some circumstances when this does not occur (Tarride et al., 2016; Wijbenga et al., 2016; Wang et al., 2018). For instance, large bone defects, such as trauma, tumor resection, and skeletal abnormalities can compromise integrative brain areas (Dimitriou et al., 2012; Wang et al., 2018), an issue with which we acquired experience and have contributed to a better understanding of the integrative brain function in health and disease (Vasquez et al., 1992, 1997, 1998; Sert Kuniyoshi et al., 2011; Viña et al., 2021).

Regarding advances in the filling of large bone defects, inorganic bovine bone (IBB), which promotes deposition and mineralization of the bone matrix, is an attractive research field in translational research (Aludden et al., 2018; Kasuya et al., 2018). Moreover, this osteoconductive material with particles of varying size preserves the space within the tissue and acts as a scaffold for deposition and mineralization of the bone matrix (Kasuya et al., 2018). It has been recommended mainly because its unlimited availability simplifies the surgical procedure (Aludden et al., 2018). But, to date and despite the scientific and biotechnological advances, there are many open questions, including its potential to interact with bone defects critical enough to preclude spontaneous and complete bone healing, including critical-sized defects.

More recently, experimental and clinical studies have focused on infrared wavelength, which seems to be more effective than the red wavelength and has also been a promisor complement for the process of critical defects (Brassolatti et al., 2018). However, irradiation of large bone defects with laser devices still needs more investigation to clear some important questions, such as the number of sessions required to achieve improvements in new therapies (Vajgel et al., 2014; Santinoni et al., 2017; Brassolatti et al., 2018). The use of low level laser therapy (LT) is considered a promising approach to improve the osteoconductive and reparative potential when combined with the biomaterial IBB (Barbosa et al., 2013; Marques et al., 2015; Acar et al., 2016; Bosco et al., 2016; de Oliveira et al., 2018). At the same time, the low-level laser therapy (LT) figures as an innovative invasive treatment of bone lesions that stimulates osteogenesis and accelerates tissue healing (Bai et al., 2021). Although the mechanisms regarding regenerative bone tissue are unclear, recent findings indicate that the light absorbed by the plasma membrane triggers a variety of biochemical reactions that activate transcription factors and modulate cell migration, mitosis, and tissue repair (Kunimatsu et al., 2018).

Considering that low-level laser therapy may improve the osteoconductive and reparative potential of the biomaterial IBB (de Oliveira et al., 2018), this study was designed to evaluate how many sessions of LT are required to improve the effects of IBB filling of large bone injuries in the head.

## Materials and Methods

### Ethical Aspects and Sample

This animal study was previously approved by the Ethics Committee on Animals Use of the Federal University of Espírito Santo (CEUA-UFES #010/2014). The procedures complied with those established by the European Community guidelines for animal experimentation. A total of 42 male Wistar rats *(Rattus norvegicus albinus)* with body weight between 250 and 300 g, were kept in individual cages at 22 ± 2°C, 50 ± 10% relative humidity, 12 h light/dark cycles, with free access to water and standard chow.

### Study Design

The animals were randomly assigned to seven groups of six animals each according to the number of laser therapy and evaluation sessions 0 (non-irradiated; Control group), 4, 7, or 11 and with the evaluation periods of 15- or 30-days post-surgery: 0LT15d and 0LT30d; 4LT15d and 4LT30d; 7LT15d and 7LT30d; 11LT30d. This last group could only be evaluated at 30 days since the last session occurred on the 21st day.

### Surgical Procedure

**FIGURE 1:**
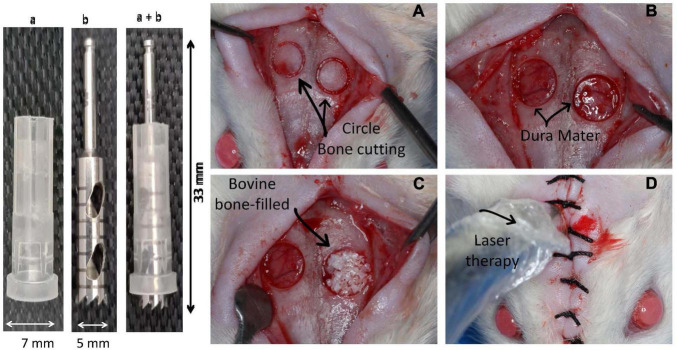
Device and approach used to induce bone damage in the head of Wistar rats. In the left side is shown the device used to remove a circular 5-mm-diameter and 2-mm-deep bone area on the top of a rat. The device was constructed by using a 7-mm-diameter protective plastic tube (“a”) covering a metallic diamond bur (“b”) and the mounted device (“a” + “b”). In the right side is illustrated the sequential steps of the surgical approach. **(A)** Shows the area delimited by the cutting device; **(B)** illustration of the integrity of the dura mater after removing the bone; **(C)** right side cavity filled with inorganic bovine bone (IBB); **(D)** radiation of the cavities with a low-level laser device.

After aseptic preparation, animals were anesthetized by intraperitoneal injection of 50 mg/kg ketamine (Instituto BioChimico Industria Farmaceutica, Rio de Janeiro, Brazil) and 5 mg/kg xylazine (Syntec do Brazil, Santana do Parnaiba, Brazil). After trichotomy of the dorsal cranium of each animal and antisepsis of the skin and surrounding hair with 2% chlorhexidine digluconate, a longitudinal incision of approximately 2.5 cm was made along the sagittal suture to expose the calvaria bone cortex. Two circular critical sized defects, approximately 2 mm from each other, were prepared on the right and left sides of the parietal bone without damaging the surrounding sutures (sagittal, coronal, and lambdoid) (Figure 1). Craniotomy was performed with a 5-mm external diameter trephine bur (W&F Cirurgicos, Barueri, Brazil) mounted in a 20:1 low-speed hand piece at 1,500 rpm (Kavo, Joinville, Brazil) and under abundant irrigation with sterile 0.9% saline solution. Both external and internal cortical were cut without injury to the dura mater since a plastic device was coupled to the bur to limit the cutting depth (Figure 1). In all groups, the right cavity spontaneously filled a blood clot, while the left cavity was filled with 14.7 μL of IBB (Bio-Oss^®^ 0.25 – 1 mm granules, Geistlich Pharma, Wolhusen, Switzerland) (Hallman and Thor, 2008).

The periosteum and scalp were sutured in a single plane with interrupted stitches made with 3.0 silk thread (Johnson & Johnson, São Paulo, Brazil) and the rat skin was marked with a dermographic pen to facilitate the application of the laser. Next, animals were kept in the prone position in their respective cages and under supervision for anesthesia recovery. A single dose of antibiotics (Enrofloxacin 2.5% 10 mg/kg, Vencofarma, Londrina, Brazil) was administered subcutaneously. To control postoperative pain over the next 3 d, 30 drops of analgesic (Tylenol, 200 mg/mL, Janssen-Cilag Farmaceutica, São Jose dos Campos, Brazil) were diluted in each 200 mL of drinking water.

### Laser Therapy

The transcutaneous LT protocol began immediately after the surgery and followed 4, 7, or 11 sessions at 48 h intervals (de Oliveira et al., 2014). The animals were sedated in an inhalation chamber by using a cotton pad soaked with 2.5 mL of anesthetic (Isoflurane^®^, Instituto BioChimico Industria Farmaceutica, Rio de Janeiro, Brazil). A gallium-aluminum-arsenide (GaAlAs) diode laser (Laser Duo, MMOptics, São Carlos, Brazil) was applied at a continuous infrared wavelength of 808 nm, power output of 100 mW, ø ∼ 3 mm^2^, 60 s, and energy density of 200 J/cm^2^ per session (Cunha et al., 2014; de Almeida et al., 2014; Moreira et al., 2018). Irradiation was conducted with the laser tip in contact with the skin at each cavity (right and left) (Figure 1D). After the postoperative periods of 15 and 30 days, the animals were euthanized by the same operator with a lethal dose of ketamine (150 to 225 mg/kg) and xylazine (15 to 30 mg/kg). Samples of the calvarial grafted areas were collected and immediately fixed in 10% neutral buffered formalin (Histopot-Serosep, County Limerick, Ireland).

### Histological and Histomorphometric Analyses

Calvarial samples were decalcified in a 10% formic acid with daily changes for five consecutive days, sectioned in the sagittal direction (at the greatest CSD diameter) and then subjected to a series of baths for histological processing. Serial sections with a 6-μm thickness were obtained and stained with standard hematoxylin and eosin (H&E). A photomicrograph of one section representing the central area of each CSD was obtained with a microscope coupled to a digital camera (Leica DM50 + ICC50 HD, Leica Reichert & Jung Products, Wetzlar, Germany) with 10 × magnification. For the histomorphometric analysis, the total area and the areas of newly formed bone were marked by a previously trained and blinded evaluator with the aid of imaging software (Image J, National Institute of Mental Health, Bethesda, MD, United States). The findings were calculated as percentages of mineralized tissue to the total area.

### Microtomographic Analysis

Calvarial bone blocks were scanned at the Laboratory of Microscopy and Biological Image of the Federal University of Paraiba with a computed microtomography machine (SkyScan 1172, Bruker, Kontich, Belgium) set with the following parameters: 80 kVp, 120 μA, 0.5 Al filter, 14 μm voxel size, 0.3° rotation step, 3 frames, and a 180° rotation. The cross-section slices were reconstructed by using beam-hardening artifact correction of 20% (NRecon, Bruker) and volume realignment (DataViewer, Bruker). After the delimitation of the entire CSD area (5-mm-diameter circular-shaped structure and the total calvarial thickness), the following bone parameters were quantified: bone volume (BV), trabecular number (Tb.N), and thickness (Tb. Th) (CT Analyzer, Bruker).

### Statistical Analysis

D’Agostino-Pearson, Shapiro-Wilk, and Kolmogorov-Smirnov tests were applied to assess the normal distribution of the data, which was confirmed by the agreement of at least two tests. Then, groups were statistically compared using one- or two-way analysis of variance (ANOVA) and Tukey’s *post hoc* multiple comparisons at a significance level of *p* < 0.05 (Prism 8.0, GraphPad Software, San Diego, CA, United States).

## Results

### Histology of Clot-Filled Cavities

In the 0LT subgroups, it was observed in typical images that regardless of the subgroup, bone tissue regeneration was not complete at the observation periods. After 30 days, less fibrous tissue was observed. A small amount of mineralized bone matrix at the margins and in some CSD central spots, a large amount of connective tissue with collagen fibers parallel to the periosteum and limited osteoblastic activity were observed.

As illustrated by typical images in Figure 3, differently from the Control group, all experimental groups showed similar characteristics such as greater deposition and mineralization of bone matrix both at the margins and in the CSD central area, mineralized areas joined together by bridges of bone tissue toward the central area with a greater tendency for complete closure, more intense osteoblastic activity, and a lesser amount of connective tissue at the two evaluation periods.

**FIGURE 2:**
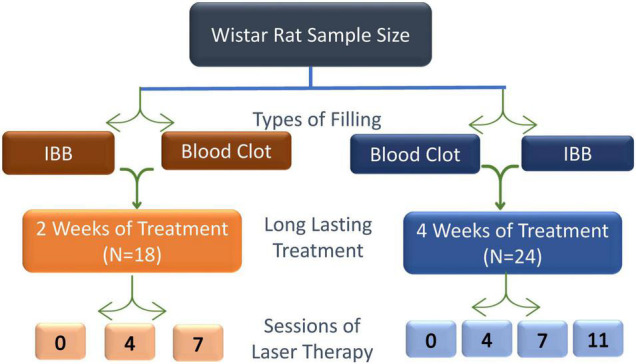
Schematic design used to achieve the aim of this study, the effectiveness, and the number of sessions of low-level LT required to improve the recovery of bone injury. Sessions were scheduled to occur at intervals of 48 h.

**FIGURE 3:**
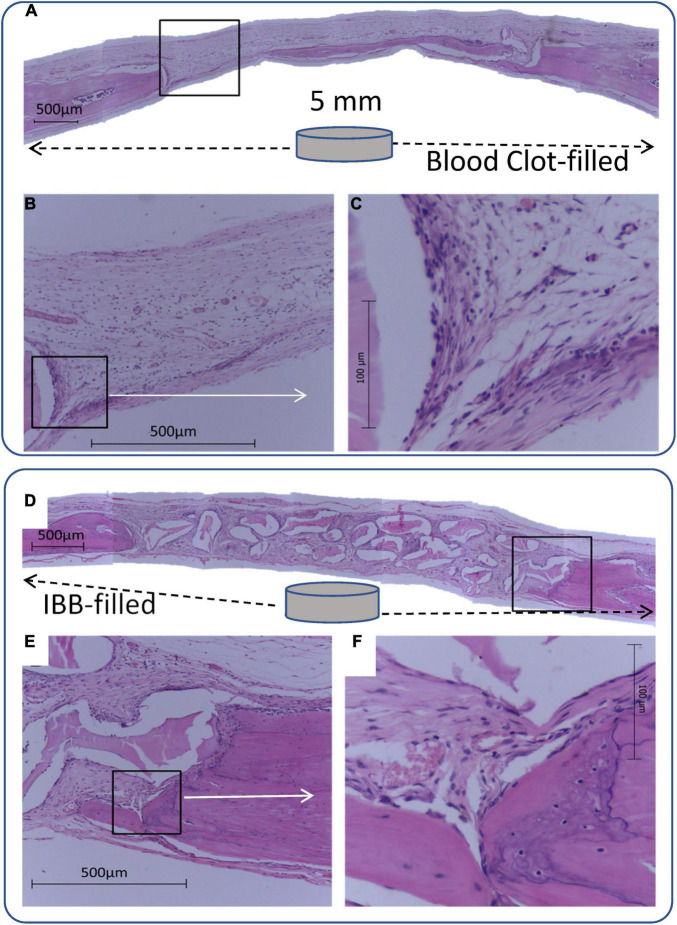
Panoramic view of a 5-mm-diameter defect produced in a rat. Cavity defects were filled with blood clot **(A–C)** or inorganic bovine bone **(D–F)**, associated with 7 sessions of laser irradiation can be compared. Images were magnified (C and F) to highlight the recovery process, suggesting an improvement when the filling of the cavity is through IBB, as indicated by intense osteoblastic and fibroblastic activity at the margins with deposition of bone matrix and collagen fibers.

### Histology of IBB-Filled Cavities

Regardless of the group, the biomaterial graft induced bone formation throughout the cavities and with a thickness similar to the surrounding bone tissue.

In the 0LT15d (Control group) filled with IBB, there was connective tissue with parallel collagen fibers with the presence of osteoblasts and new blood vessels. Osteoclasts around the biomaterial particles were found in some areas and a few mineralized spots were observed at the CSD margins. After 30 days, more pronounced matrix mineralization around and between IBB particles was observed in addition to moderate mineralization in the central CSD area.

Figure 3 (bottom panel) illustrates panoramic views of the effect of IBB filling in a 7LT15d animal. All groups presented similar findings such as the great amount of well-organized connective tissue with parallel collagen fibers, intense osteoblastic activity, and numerous blood vessels. At the 15-days evaluation, a moderate increase of mineralized areas at the margins and sparse islands of mineralization at the margins and between the particles were found (Figures 3E,F). After 30 days, a greater amount of mineralized matrix was observed at the margins and toward the CSD center. Moreover, mineralized matrix was observed between and within some porosities of biomaterial particles.

**FIGURE 4:**
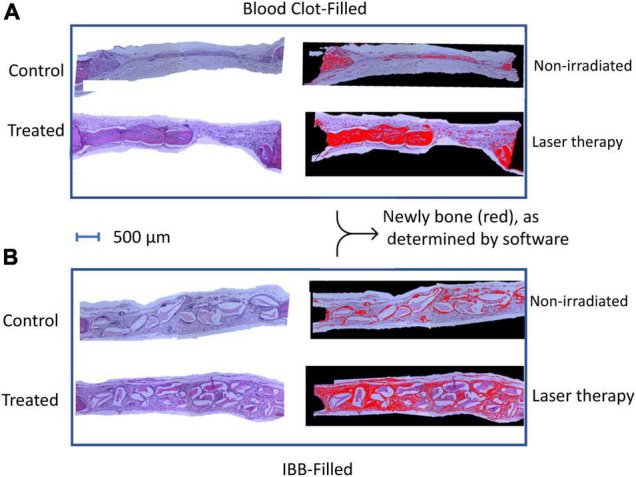
Photomicrographs of representative images of hematoxylin-eosin (HE) sections of both subgroups, the blood clot **(A)**, and the IBB filled cavities **(B)**. Images were delimited with the aid of imaging software to compare the areas of newly formed bone (in red color).

Figure 4 shows typical images from a rat of seven sessions of LT on the clot-filled cavity and compared with a non-irradiated Control rat (top panel). In the bottom panel is shown a typical image of a 7LT15d rat with IBB-filled and compared with a 0 non-irradiated animal (0LT15d). Images were delimited with the aid of imaging software to compare the areas of newly formed bone (in red color).

**FIGURE 5:**
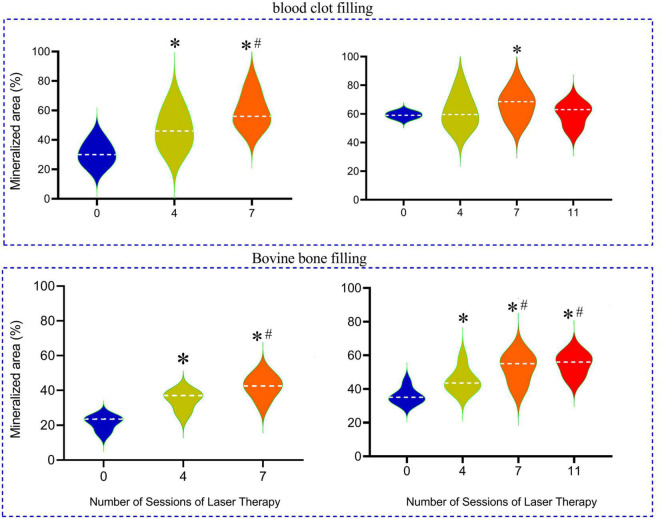
Percentage of mineralized bone produced in the cavities that were filled with inorganic bovine bone (IBB) in comparison with blood clot-filled groups, mainly when associated with 4 to 11 sessions of LT and when compared with the non-irradiated group (0). The horizontal dashed line into the violin repress represents the mean values for each group. *p < 0.05 compared with the mean values of the Control group; ^#^*p* < 0.05 compared with the 4 sessions group.

The percentage of mineralized areas which was facilitated by clot, a natural regenerative material, or by IBB in the cavities and analyzed at 15 days post-surgery is summarized in the violin graphs of Figure 5.

In the group of rats with cavities filled by a blood clot, the percentage of mineralized area in the reparative process of bone tissue in a period of 2 weeks averaged 30 ± 4% in the Control group (0LT15d) and was significantly augmented in the 4LT15d group (47 ± 5%) and even higher in the 7LT15d group (*p* < 0.05) reaching a mean of 58 ± 5%. When the period of time is extended to 4 weeks, we did not observe a so great improvement, probably due to the fact that the blood clot, by itself, had a great reparative effect, as indicated by the high percentage of mineralization (59 ± 6%) observed in the Control group (0LT30d). Note that the violin graph of this group was of the lest variability, indicating that all animals of this group were very similar (low variability). The 4LT30d group exhibited similar values (62 ± 5%) when compared to the Control values but significantly improved effect as compared with the group of 4LT15d. The group of seven sessions into the 30 days (7LT30d) showed an additional improvement to 66 ± 4%, but even increasing the number of sessions to 11 in the sub-group 11LT30d, the values returned to a level (60 ± 3%) similar to the Control group.

In the subgroups in which IBB was used as biomaterial to fill the cavities, the bone mineralization was 22 ± 3% in the Control group (0LT15d) and was similarly augmented in the 4LT15d (37 ± 3%; *p* < 0.05) and 42 ± 3% in the 7LT15d group. Still, in this group of IBB-filled material with the analysis done 30 days post-surgery, the Control non-irradiated group (0LT30d) showed an area 36 ± 3% of mineralization and it was statistically improved in the 4LT30d group (45 ± 3%) and higher when the number of sessions increased (7LT30d and 11LT30d groups), reaching similar averages of 52 ± 3% and 53 ± 3%, respectively (*p* < 0.05).

**FIGURE 6:**
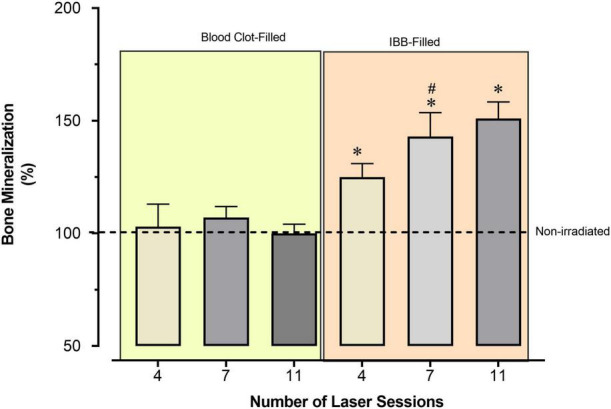
Average percentage of bone mineralization caused by the type of cavity filling and the required number of sessions of LT, compared to the reference values observed in the Control group, expressed as 100% (horizontal dashed line). Values indicate the mean and the mean standard error. *p < 0.05 vs. Control group; ^#^*p* < 0.05 vs. the previous time-point of the same group. Statistical analysis: two-way ANOVA for *N* = 6 rats per group.

To avoid the confounder factor of differences in the referential values observed in each of the four Control groups, we decided to provide an additional analysis of the data, which is presented in Figure 6. In the additional analysis we show the average values of bone mineralization caused by the type of cavity filling and the required number of LT sessions. These values were compared to the reference levels of the Control groups, which were expressed as 100%. In this way, IBB-filled provided much better reparative effects than those observed in the blood clot-filled ones. The data also show that 4 sessions is sufficient to increase generation of new bone and that there is no benefit going longer than 7 sessions (Figure 6).

### Microtomographic Analysis

**FIGURE 7:**
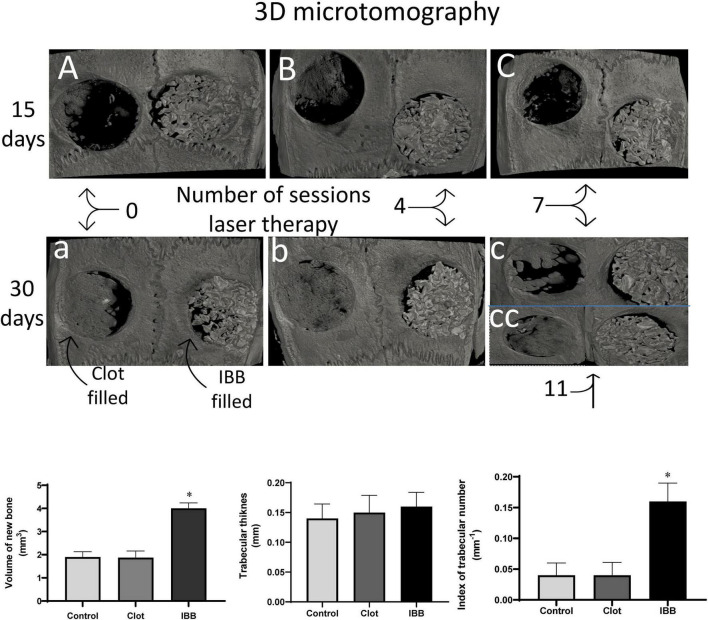
Typical 3D microtomographic images, comparing blood clot-filled (left) with IBB-filed (right) cavities and comparing the analysis at 15 days (A, B, and C) with 30 days (a, b, c, and cc). Figure illustrates the effects of the number of sessions of LT, which in the group of 30 days was extended to a group of 11 sessions (cc). At the bottom are shown the qualitative data obtained by the microtomographic analysis. Values are mean ± Standard error of the mean. *p < 0.05 vs, Control and clot-filled groups. Statistical analysis: two-way ANOVA for *N* = 6 rats per group.

The results described above are corroborated by the qualitative and quantitative analysis provided by three-dimensional microtomography (Figure 7). As seen in the typical images (top panel), the defect area of the Control animal (blood clot- or IBB-filled) lacks new bone, when examined 2 weeks later (A) and shows some improvement when examined at 4 weeks later (“a”). In contrast, the LT irradiation of cavities filled with blood and bone showed gradual improvements (B and C) and with a remarkable regenerative process (neoformation of bone tissue) when the cavities were filled with IBB and concurrent LT (“a”).

Column graphs at the bottom of Figure 7 show descriptive statistics of 3 parameters measured by three-dimensional microtomography. An amount of 4.0 ± 0.2 mm^3^ of new bone was found in the cavities filled with IBB, indicating that this biomaterial combined with concurrent LT results in approximately twofold that amount formed in cavities filled with blood clot (1.872 ± 0.2 mm^3^, *p* < 0.05). A similar volume was observed in the Control group (1.9 ± 0.2 mm^3^, *p* > 0.05). The data about trabecular thickness did not show significant differences among groups, indicating that the increased volume was not due to augmented thickness of the trabeculae. In fact, the next analysis showed an increase of approximately threefold in the trabecular number in the group IBB-filled (0.160 ± 0.02 mm^–1^), compared with the blood clot-filled group (0.0387 ± 0.01 mm^–1^, *p* < 0.05), which was remarkably close to that observed in the Control group (0.04 ± 0.01 mm^–1^). Therefore, one of the possible mechanisms by which LT speeds up and improves the recovery process of critical bone defects seems to include the augmented trabecular number and volume of new bone.

## Discussion

The present study was motivated by the challenge to contribute to the knowledge about the reparative process of large calvarial defects, and then, to create novel approaches to obtain a fast, non-invasive, not painful, not expensive, and an efficient approach to repair critical bone defects. The quantitative data generated can be expressed as summarized here. IBB-filled large defects provided a faster and better recovery of bone function than the blood clot-filled alternative. The formation of new bone into the cavities gradually increased when the bone injury was filled with IBB, and it was greatly improved when the grafting was combined with sessions of LT. The new bone formed into the cavity increased approximately 25%, 50%, and 55%, respectively, with 4, 7, and 11 sessions of LT and compared to referential Control values obtained in IBB-filled but not irradiated with low-level laser, i.e., combining the use of IBB with seven sessions of LT seems to be an optimal approach to greatly improve the recovery of calvarial defects. It would be important to verify if the optimal effects of seven sessions of LT on bone mineralization are also observed in cavities filled with other biomaterials such as the bioactive glass particles which have been used in *in vitro* studies from other investigators (Alves et al., 2017).

To avoid possible bias and inappropriate analysis and consequent conclusion, our protocols followed rigorous procedures as follows. The development of a device (Figure 1) allowed us to produce exactly the same diameter and depth of the cavities in all animals. Moreover, the effectiveness of each kind of filling was possible to be precisely evaluated in the animals of the three main groups (non-irradiated Control and irradiated and examined at 15 vs. 30 days). In addition, all animals received the same grafting material from the same manufacture (Bio-Oss®; Geistlich Pharma, Wolhusen, Switzerland). Despite the cavity requiring a very small amount of grafted material, we are confident that in each cavity of each animal, the volume of the biomaterial was exactly the same in all of them (14.7 μL), which was reliable due to the use of a device that was used in all animals. Therefore, we believe that important sources of bias commonly occurred in this type of study were prevented, and this is the reason of our interpretation that the differences in the results are due to the therapeutic characteristics of the biomaterial, accompanied by the irradiation with low-level LT.

The preparation of two critical sized defects with both experimental and control sites or blood clot-filled and IBB in the same rat calvaria (paired split design) was provided as a goal to reduce possible bias caused by the variability between different animals (Vajgel et al., 2014). Despite the lack of consensual opinion, it is assumed that a 5-mm-diameter defect seems to avoid complete closure with newly formed bone in a short period of time (de Almeida et al., 2014; Vajgel et al., 2014; Brassolatti et al., 2018).

In this study, the histological analysis of the cavities filled with a blood clot showed that bone formation was not complete after 30 d post-surgery. Regarding the fact that this is a controversial issue (Hawkins and Abrahamse, 2005; Cunha et al., 2014; Brassolatti et al., 2018; Noba et al., 2018), in the present study, we tried to use consensual dosimetric parameters (wavelength, application time, and energy density per session) that have been reported by previous prestigious investigators (Cunha et al., 2014; de Almeida et al., 2014; Moreira et al., 2018.) Considering the premise that a beneficial effect of LT on bone tissue formation in 5-mm-diameter critical sized defects has been already evidenced and that the number of LT sessions used to be directly related to the final amount of stimulation delivered to the tissue and could interfer in the final result of the process (Brassolatti et al., 2018). The present investigation specifically focused on the number of LT sessions required to significantly improve the recovery process.

The histomorphometry analysis has been considered the “gold standard” for the evaluation of bone healing and quantification of cell activity in terms of percentage of newly formed bone (Pinheiro et al., 2012; Acar et al., 2015). Furthermore, using this technique one can measure *ex vivo* bone volume and represent a viable alternative to qualitative and quantitative analyses in the research field of repairment of hard tissues (Schmidt et al., 2003; Schambach et al., 2010). The microtomographic analysis was able to accurately identify newly formed bone in critical sized defects at both 15- and 30-days evaluations. The use of high-resolution images and statistical procedures of normalization of reference values (Control group), it was possible to measure trabecular structures of the calvarial defects, and to detect differences in the volume of newly formed bone and in the trabecular number between, when comparing the IBB group with a bone clot or the Control groups. These findings are not a surprise because they are in agreement with the results obtained with the analysis using HE staining shown in Figure 6. Although we did not find significant differences in the average values of trabecular thickness among groups, the lack of difference does not seem to be due to technical aspects interfering in the analysis of microtomographic parameters (Schambach et al., 2010) because we acted with maximal rigor and the procedures were performed by skilled researchers in all steps (Chatterjee et al., 2017). Moreover, some authors suggest that microtomography presents limited sensitivity to identify differences in bone tissue after only 2- and 4-weeks post-surgery since the matrix is not completely mineralized at that time (Schambach et al., 2010; Ramalingam et al., 2016; de Oliveira et al., 2018).

The histomorphometric analysis of clot-filled cavities indicates that a greater number of LT sessions accelerated bone tissue formation during the initial stages of the bone repair process, which is stabilized later. Thus, LT seems more effective at the initial phase of intense cell proliferation following deposition and mineralization of bone matrix (Ozawa et al., 1998; Matsumoto et al., 2009; Marques et al., 2015). However, our results agree with previous studies showing that LT increases the formation of bone tissue in both clot- and IBB-filled cavities (Rasouli Ghahroudi et al., 2014).

Considering that the cavities were grafted with IBB and analyzed after 15 days, we did not expect that a higher number of LT sessions would benefit a better outcome, and in fact, we did not observe important differences in bone formation between 4LT15d and 7LT15d sub-groups. In contrast, after 30 days we found significant differences when comparing the percentage of mineralization in 7LT30d and 11LT30d sub-groups and, in comparison, with Control groups (non-irradiated). The fact that the 4LT30d sub-group was not significantly different from 0LT30d sub-group could indicate that a minimum number of LT sessions is needed to increase bone tissue formation. Conversely, the absence of a significant difference between 7LT30d and 11LT30d sub-groups suggests that the amount of mineralized tissue tends to stabilize after 7 LT sessions.

Our findings indicated that beneficial effect of LT in IBB-filled cavities evaluated 15 and 30 days post-surgery. However, one could argue that this could be a misinterpretation because in the group of 30 days one of the Control groups had levels of mineralization completely different from the other sub-groups. To avoid this confounding factor, we decided to normalize and to use the most accepted solution when one deals with comparison of groups exhibiting marked and unexpected differences in the basal or control values. Surprisingly (see Figure 6), we found remarkably interesting results that led us to propose changes in our initial hypothesis. What we found was that the differences between the experimental sub-groups (number of sessions) and the non-irradiated Control group are not influenced by the number of sessions with LT in blood clot-filled cavities. In contrast, in IBB-filled cavities groups it was observed that the percentage of mineralization is clearly associated with the increase of LT sessions, and that the magnitude of the response does not recommend using more than seven sessions of LT is an exciting result.

Other authors have been pioneers in the demonstration of the beneficial effects of combining IBB, which has similarity to human bone, with low-level LT (Cunha et al., 2014). The present data provides a contribution to this research field, by focusing on the number of LT sessions required to speed and amplify the effects of this combination. Despite our exciting results using IBB, other bioactive materials such as bioactive glass particles (Alves et al., 2014) could also be tested. We are confident about our results because we were challenged to perform a three-dimension microtomographic analysis, which has been an important approach in this research field (Alves et al., 2014). That procedure showed that the combination seven sessions of LT with IBB resulted in a significant increase in the volume of new bone formation (∼twofold) and an increase in the trabecular number (∼threefold). However, other avenues have been opened with this translational research and need to be pursued before moving up to clinical trials.

One exciting contribution to the mechanisms involved with the osteogenic and mineralization of cellular and extracellular matrix in the recovery of calvaria bone defects was recently provided by Alves et al. (2014). Through *in vitro* manipulation of newborn osteogenic cells those authors revealed some conditions favoring immunolabeling proteins as important contributors to the osteoblastic activities and differentiation and formation of mineralized matrix (Alves et al., 2014). Based on this scenario, we conclude that our findings show that IBB-filled cavities irradiated during seven sessions of LT could favor the regenerative process of great-sized defects and challenge other investigators to join us in the search for rational solutions in the fighting against critical calvaria defects.

## Conclusion

The present study is related with approaches to achieve rapid and effective repair of critical bone defects by using grafting inorganic material and low-level LT. Our data demonstrated that critical calvaria defects filled with IBB produces have much better reparative results than those filled with blood clot, and that the combination of these techniques with low-level LT accelerates and increases the bone healing. We determine the number of sessions of laser therapy required to obtain minimal and maximal effects in the regenerative process of bone critical defects. The data revealed that a maximum benefit is obtained using IBB combined with seven sessions of low-level LT. Therefore, the present results greatly contribute to the expected promotion of better conditions for those suffering from bone trauma- and other cause-related critical injuries. The present study and conclusions may provide new insights and motivate clinical trials in this research field.

## Data Availability Statement

The raw data supporting the conclusions of this article will be made available by the authors, without undue reservation.

## Ethics Statement

The animal study was reviewed and approved by Ethics Committee in the Use of Animals, Federal University of Espirito Santo, Brazil.

## Author Contributions

MS, DS, RB, SM, and EV made substantial contributions to the concept and design of the study and interpretation of the data. MS conducted the experiments and provided acquisition and analysis. KR, FS, and ED performed the microtomographic analysis. LL and EV processed the spreadsheet data, constructed the graphs, and provided statistical analysis. TP contributed with the concept, analysis, and production of Figure 6 and collaboration in the illustration of microtomographic images of a new graph suggested by the reviewers. EV coordinated the critical revision of the manuscript and provided the final version. All authors contributed to the article and approved the submitted version.

## Conflict of Interest

The authors declare that the research was conducted in the absence of any commercial or financial relationships that could be construed as a potential conflict of interest.

## Publisher’s Note

All claims expressed in this article are solely those of the authors and do not necessarily represent those of their affiliated organizations, or those of the publisher, the editors and the reviewers. Any product that may be evaluated in this article, or claim that may be made by its manufacturer, is not guaranteed or endorsed by the publisher.
